# Anti-Inflammatory Potential of 1-Nitro-2-Phenylethylene

**DOI:** 10.3390/molecules22111977

**Published:** 2017-11-15

**Authors:** Michelle A. Sugimoto, Márcia de Jesus Amazonas da Silva, Larissa Froede Brito, Rosivaldo dos Santos Borges, Flávio Almeida Amaral, Ana Paula de Araujo Boleti, Maritza Echevarria Ordoñez, Jose Carlos Tavares, Lirlandia Pires Sousa, Emerson Silva Lima

**Affiliations:** 1 Laboratory of Inflammation Signaling, Department of Clinical Analysis, Faculty of Pharmacy, Federal University of Minas Gerais, Belo Horizonte 31270-901, MG, Brazil; adrianeamantea@gmail.com (M.A.S.); larissafroede@yahoo.com.br (L.F.B.); lipsousa72@gmail.com (L.P.S.); 2 Laboratory of Biological Activity, Faculty of Pharmaceutical Sciences, Federal University of Amazonas, Manaus 69067-005, AM, Brazil; marciajas24@gmail.com; 3 Nucleus of Studies and Selection of Bioactive Molecules, Institute of Health Sciences, Federal University of Pará, Belém 66075-110, PA, Brazil; lqfmed@gmail.com (R.d.S.B.); apboleti@yahoo.com.br (A.P.d.A.B.); echevarriamaritza8@gmail.com (M.E.O.); 4 Department of Physiology and Biophysics, Institute of Biological Sciences, Federal University of Minas Gerais, Belo Horizonte 31270-901, MG, Brazil; dr.famaral@gmail.com; 5 Laboratory of Research in Drugs, Department of Biological Sciences and Health, Federal University of Amapá, Macapá 68903-419, AP, Brazil; jctcarvalho@gmail.com

**Keywords:** 1-nitro-2-phenylethylene, anti-inflammatory activity, TNF-α production

## Abstract

Inflammation is a reaction of the host to infectious or sterile stimuli and has the physiological purpose of restoring tissue homeostasis. However, uncontrolled or unresolved inflammation can lead to tissue damage, giving rise to a plethora of chronic inflammatory diseases, including metabolic syndrome and autoimmunity pathologies with eventual loss of organ function. Beta-nitrostyrene and its derivatives are known to have several biological activities, including anti-edema, vasorelaxant, antiplatelet, anti-inflammatory, and anticancer. However, few studies have been carried out regarding the anti-inflammatory effects of this class of compounds. Thereby, the aim of this study was to evaluate the anti-inflammatory activity of 1-nitro-2-phenylethene (NPe) using in vitro and in vivo assays. Firstly, the potential anti-inflammatory activity of NPe was evaluated by measuring TNF-α produced by human macrophages stimulated with lipopolysaccharide (LPS). NPe at non-toxic doses opposed the inflammatory effects induced by LPS stimulation, namely production of the inflammatory cytokine TNF-α and activation of NF-κB and ERK pathways (evaluated by phosphorylation of inhibitor of kappa B-alpha [IκB-α] and extracellular signal-regulated kinase 1/2 [ERK1/2], respectively). In a well-established model of acute pleurisy, pretreatment of LPS-challenged mice with NPe reduced neutrophil accumulation in the pleural cavity. This anti-inflammatory effect was associated with reduced activation of NF-κB and ERK1/2 pathways in NPe treated mice as compared to untreated animals. Notably, NPe was as effective as dexamethasone in both, reducing neutrophil accumulation and inhibiting ERK1/2 and IκB-α phosphorylation. Taken together, the results suggest a potential anti-inflammatory activity for NPe via inhibition of ERK1/2 and NF-κB pathways on leukocytes.

## 1. Introduction

*Aniba canelilla* (H.B.K.) Mez [syn. *Aniba elliptica* A. C. Sm., *Cryptocarya canelilla* Kunth] (EOAC), commonly known as “casca preciosa” (precious bark), is an aromatic plant abundant in the Amazon region, and belongs to the Lauraceae family. In folk medicine, the decoction of bark wood is used as an antispasmodic, a digestive stimulant, and a carminative [1,2,3]. Indeed, *A. canelilla* bark oil exerts cardiovascular effects, causing hypotension and bradycardia in normotensive rats [4], as well as presenting antioxidant properties [5]. 

**Figure 1:**
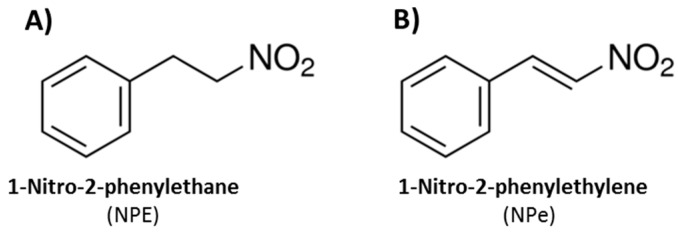
Chemical structures of (**A**) 1-nitro-2-phenylethane (NPE), the main component of *Aniba canelilla* essencial oil; and (**B**) synthetic 1-nitro-2-phenylethylene (NPe).

The essential oil of *A. canelilla* is rich in 1-nitro-2-phenylethane (NPE, Figure 1A), the first nitro compound isolated from plants [6]. NPE is considered to be the odoriferous principle of leaf, bark, and trunk wood of *A. canelilla*, responsible for the cinnamon scent [6,7]. The content of NPE in the plant depends on the season, been more abundant in the rainy than in the dry period [8]. NPE has been shown to exert antinociceptive [9] and, vasorelaxant [10,11] effects in vivo. Furthermore, a recent study has suggested that NPE also has anti-inflammatory activity, as demonstrated by its ability to inhibit paw edema induced by dextran and carrageenan in rats and by croton oil in mice [12].

NPE assumes different conformations due to its sp3 carbon atoms. A conformational restriction by substitution of the alkane for the alkene moiety allows the formation of 1-nitro-2-phenylethene (NPe, Figure 1B), also named 1-((*E*)-2-nitro-vinyl)-benzene or β-nitrostyrene [13]. NPe could have increased drug potency compared to NPE, since the removal of torsional flexibility is known to reduce the entropic penalty of binding to the target [14,15].

Inflammation is a reaction of the host to infectious or sterile tissue damage which aims to eliminate pathogens, clear damaged host cells and restore tissue homeostasis [16,17]. However, uncontrolled or unresolved inflammation can lead to tissue damage, giving rise to a plethora of chronic inflammatory diseases [17,18]. The acute inflammatory response can be divided into initiation and resolution stages [19]. During the early phase of inflammation, production of inflammatory mediators promotes leukocyte accumulation and survival in the inflammatory site, while the resolution of inflammation depends on proper clearance of migrated leukocytes, mainly via neutrophil apoptosis and their removal (efferocytosis) by tissue macrophages [17].

Recognition of microbial antigens plays a crucial role in host defense, and relies on the detection of conserved molecular patterns that are essential products of microbial physiology, such as lipopolysaccharides (LPS) of Gram-negative bacteria and peptidoglycans of Gram-positive bacteria [20]. LPS binds to toll-like receptor (TLR)-4 on the surface of macrophages, triggering the activation of signal transduction pathways, including the well-characterized NF-κB and mitogen-activated protein kinase (MAPK)-dependent transcription factors, which induce the expression of many of the pro-inflammatory cytokines and immune mediators [21,22,23]. LPS-activated NF-κB has been shown to be critically involved in the transcriptional regulation of the tumor necrosis factor-α (TNF-α), interleukin (IL)-6, and IL-1 genes in macrophages [22]. In resting cells, NF-κB dimers are sequestered to the cytoplasm and maintained inactivated by reversible association with its inhibitor IκB or unprocessed forms of cytoplasmic p50/p105 (NF-κB1) and p52/p100 (NF-κB2) [24,25,26]. NF-κB activation in response to proinflammatory stimuli is regulated by IKK, which phosphorylates IκB and promotes its proteasome degradation and the release of NF-κB for nuclear translocation and gene transcription activation [21]. Activation of the MAPK/ERK pathway by LPS has also been shown to regulate the production of pro-inflammatory cytokines, such as TNF-α, in murine macrophages [27].

In this study, we explored the potential anti-inflammatory effects of NPe, which reduced the production of TNF-α by human macrophages stimulated with LPS, and prevented neutrophil accumulation, in vivo, in a model of acute pleurisy. Mechanistically, our data indicate that NPe can decrease the activation of ERK1/2 and NF-κB inflammatory pathways, while further studies are needed to fully elucidate this mechanism of action.

## 2. Materials and Methods

### 2.1. Synthesis of NPe

**Figure 2:**
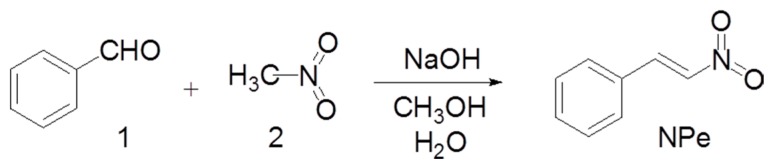
Chemical synthesis of 1-Nitro-2-phenylethylene (NPe).

NPe was synthesized in the Pharmaceutical Chemistry Laboratory at the Universidade Federal do Pará employing the Claisen–Schmidt procedure, as previously described (Figure 2) [13,28,29]. Briefly, benzaldehyde (1) and nitromethane (2) were used as reactants (0.02 and 0.024 M, respectively) in methanol. The product was ‘one-pot’ converted with 89–92% yield by using 0.1 M NaOH in water at 0–10 °C. The precipitate was filtered out and dried under vacuum to give a solid product. NPe was then crystallized in ethanol as a pale yellow solid. The final product was identified by FT-IR and NMR (^1^H- and ^13^C-NMR) spectroscopic techniques and compared with literature data [13,30]. IR νmax 1600, 1550, 1498, 1375 cm^−1^; ^1^H-NMR (CD_3_SOCD_3_, 200 MHz) δ 7.2–7.3 (d, 2H), 7.35–7.4 (d, 2H), 7.55 (d, 1H), 7.7 (d, 1H), 7.9 (d, 1H); ^13^C-NMR (CD_3_SOCD_3_, 50 MHz) δ 127.52, 128.84, 129.69, 136.82, 137.99, 140.96.

### 2.2. Mice and Ethics

All procedures described here had prior approval from the Ethics Committee in Animal Experimentation of the Universidade Federal de Minas Gerais (CEUA/UFMG, Protocol number: 83/2015). Male wild-type (WT) BALB/c mice (8–10 weeks old) were obtained from the Biosciences Unit of Institute of Biological Sciences (Belo Horizonte, MG, Brazil). Mice were housed under standard conditions, and had free access to commercial chow and water. 

### 2.3. Drugs, Reagents and Antibodies

4α-Phorbol-12-myristate-13-acetate (PMA), dexamethasone, lipopolysaccharide (LPS, from *Escherichia coli* serotype O:111:B4), and mouse anti-β-actin (#A5316) were from Sigma Aldrich (St. Louis, MO, USA). Rabbit anti-phospho-ERK1/2 and mouse anti-phospho-IκB-α were purchased from Cell Signaling Technology (Beverly, MA, USA). Secondary anti-mouse (Sc-2005) and anti-rabbit (#7074) peroxidase-conjugate antibodies were purchased from Santa Cruz Biotechnology (Santa Cruz, CA, USA) and Cell Signaling Technology, respectively. 

### 2.4. Cell Culture and In Vitro Assays

The human monocyte-like cell line THP-1 and the murine macrophage-like cell line J774A.1 were obtained from the American Type Culture Collection (ATCC, Rockville, MD, USA). THP-1 cells were cultured in RPMI 1640 medium (Cultilab, São Paulo, Brazil), supplemented with 10% heat-inactivated fetal bovine serum (FBS, Cultilab, São Paulo, Brazil) and antibiotics (100 μg/mL streptomycin and 100 U/mL penicillin, both from Sigma-Aldrich). J774A.1 cells were cultured in DMEM (Cultilab, São Paulo, Brazil) under the same conditions. Cell cultures were maintained at 37 °C and 5% CO_2_. To evaluate the effect of NPe on viability of murine macrophages, J774A.1 cells were seeded (50,000 cells per well) in 96-well cell culture plates (BD Biosciences, Franklin Lakes, NJ, USA). Twenty-four hours later, when a confluent cell monolayer was observed, media and non-adherent cells were removed from the wells and the adherent cells were treated with NPe at concentrations from 1 to 20 μg/mL for 24 h. To evaluate the cell viability of human macrophages treated with NPe, THP-1 monocytes were seeded (50,000 cells per well) in 96-well cell culture plates (BD Biosciences, Franklin Lakes, NJ, USA), differentiated into macrophages using PMA 20 ng/mL (Sigma Aldrich, St. Louis, MO, USA), and serum-deprived with FBS 0.5% for 16 h. Subsequently, cells were either left untreated, or treated with NPe at different concentrations (1, 5, 10 and 20 μg/mL) for 2 h, and thereafter with LPS (100 ng/mL) for a further 4 h. LPS was added to the wells without removing the supernatant, which means that NPe was present during the entire time of stimulation with LPS. Crystallized NPe was dissolved in dimethyl sulfoxide (DMSO) and diluted in medium supplemented with 0.5% FBS for all assays. The concentration of DMSO ranged from 0.002 to 0.04%. Cell viability was evaluated by the MTT method [31]. Viability of murine and human macrophages was tested using untreated cells and LPS-treated cells as reference, respectively. Cell viability higher than 90% was considered nontoxic for both cell lines. The potential anti-inflammatory activity of NPe was evaluated by measuring the cytokine TNF-α produced by LPS-stimulated THP-1 macrophages. For this, the supernatant was collected, and TNF-α was measured using the cytokine-specific sandwich quantitative enzyme-linked immune-sorbent assay (ELISA) according to the manufacturer’s instructions (TNF-α duo set, R&D Systems).

### 2.5. LPS-Induced Pleurisy Model

To further confirm the anti-inflammatory properties of NPe, we evaluated its effect in an established model of pleurisy induced by LPS [32,33,34]. For this purpose, mice were pretreated with an injection of NPe (0.9 mg/kg, i.p.), dexamethasone (2 mg/kg, i.p.), or vehicle, comprising 2% DMSO in phosphate-buffered saline (PBS). After 1 h, mice were challenged with an intrapleural (i.pl.) injection of LPS (250 ng/cavity) from *Escherichia coli* serotype (O:111:B4) purchased from Sigma Chemicals (St. Louis, MO, USA) or PBS. Cells were recovered from the pleural cavity 7 h after LPS injection by washing with PBS containing EDTA (1 mM). Total cell counts were performed on cells harvested from the pleural cavity in a Neubauer chamber using Turk’s stain. Differential counts were performed on cytocentrifuge preparations (Shandon III) after staining with May-Grunwald-Giemsa and using standard morphological criteria to identify cell types [32,33,34]. The results are shown as the number of cells per cavity. Cell extracts were prepared for western blot analysis as previously described [32,33,34].

### 2.6. Western Blot Analysis

THP-1 cells and inflammatory cells harvested from the pleural cavity were washed with PBS and whole-cell extracts were prepared as previously described [32,33,34]. Protein concentrations were quantified with the Bradford assay reagent from Bio-Rad (Bio-Rad, Redmond, WA, USA). Equal amounts of protein (50 μg) from each group were loaded and separated by electrophoresis on denaturing 10% polyacrylamide-SDS gels and electrotransferred to nitrocellulose membranes. Membranes were blocked for 1 h at room temperature with PBS containing 5% (*w*/*v*) nonfat dry milk and 0.1% Tween-20. Afterwards, the membranes were washed three times with PBS containing 0.1% Tween-20 and then incubated overnight at 4 °C with specific primary antibody (anti-ERK1/2, anti-phospho-ERK1/2, anti-phospho-IκB-alpha) using a dilution of 1:1000 in PBS containing 5% (*w*/*v*) BSA and 0.1% Tween-20. For normalization of the bands, the same membranes were incubated for 2 h at room temperature with anti-β-actin using a dilution of 1:1000 in PBS containing 5% (*w*/*v*) BSA and 0.1% Tween-20. After washing, membranes were incubated with appropriated horseradish peroxidase-conjugated secondary antibody (1:3000). Immunoreactive bands were visualized using the ECL detection system, as described by the manufacturer (GE Healthcare, Piscataway, NJ, USA). The values of phosphorylated levels of ERK1/2 and IkB-α were quantified by using a densitometric analysis software (ImageJ, Image Processing and Analysis in Java; NIH, Bethesda, MD, USA). Changes in protein levels were estimated, and the results were expressed in arbitrary units—AU normalized to the values of β-actin in the same sample.

### 2.7. Statistical Analysis

Results are presented as the mean ± SEM. Data were analyzed by one-way ANOVA, and differences between groups were assessed using the Student-Newman-Keuls post-hoc test, except where otherwise indicated. A *p* value < 0.05 was considered significant. Calculations were performed using the prism 5.0 software program for Windows (GraphPad software, San Diego, CA, USA).

## 3. Results

### 3.1. Definition of Toxic and Non-Toxic Doses of NPe on Murine and Human Macrophages

**Figure 3:**
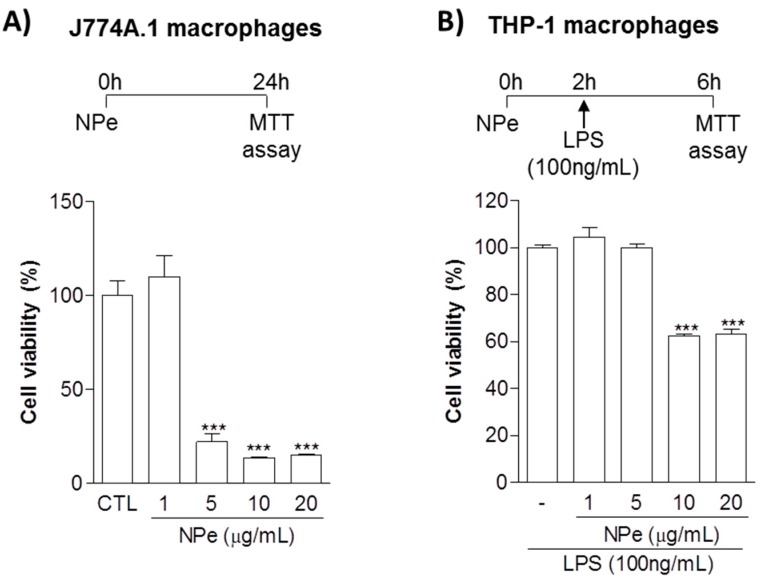
Cell viability of murine and human macrophage cell lines treated with NPe. (**A**) J774A.1 macrophage-like cells were transferred to a 96-well plate at a density of 50,000 cells per well. After 24 h, the cells were either left untreated, or treated with NPe at concentrations from 1 to 20 μg/mL for 24 h; (**B**) Human monocytic cell line THP-1 was transferred to a 96-well plate at a density of 50,000 cells per well, differentiated into macrophages and serum-deprived with FBS 0.5% for 16 h. Subsequently, cells were either left untreated, or treated with NPe at different concentrations (1, 5, 10 and 20 μg/mL) for 2 h, and thereafter with LPS (100 ng/mL) for a further 4 h. Cell viability was evaluated by the MTT method using untreated cells as a reference for viability. Data are shown as the mean ± SEM of three wells in each group. Experiments were performed at least 3 times with similar results. *** *p* < 0.001 when compared to the control group.

Initially, we aimed to define the viability of both murine and human cells exposed to NPe in order to define non-toxic doses. Murine macrophage-like cell line J774A.1 was exposed to NPe at four different concentrations (1, 5, 10 and 20 μg/mL) for 24 h. We observed cell viability below 40% when murine macrophages were treated with NPe at concentrations higher or equal to 5 μg/mL. NPe was non-toxic at the lower concentration (1 μg/mL), showing cell viability similar to the control (Figure 3A). After this initial assay, we used the same concentrations in human macrophages, using conditions that would better simulate those used in the following functional assays. To investigate cell viability of human macrophages, differentiated THP-1 macrophages were exposed to the different NPe concentrations (1, 5, 10 and 20 μg/mL) for 6 h in inflammatory conditions (under LPS stimuli). We observed cell viability below 70% when cells were treated with NPe at 10 or 20 μg/mL. As observed for the murine macrophages, NPe was non-toxic at the two lower concentrations (1 and 5 μg/mL), showing cell viability similar to the control group (Figure 3B) and, for this reason, these concentrations were used in the following assays using human macrophages.

### 3.2. Pre-Treatment of THP-1 Differentiated Macrophages with Non-Toxic Doses of NPe Has Anti-Inflammatory Effects

**Figure 4:**
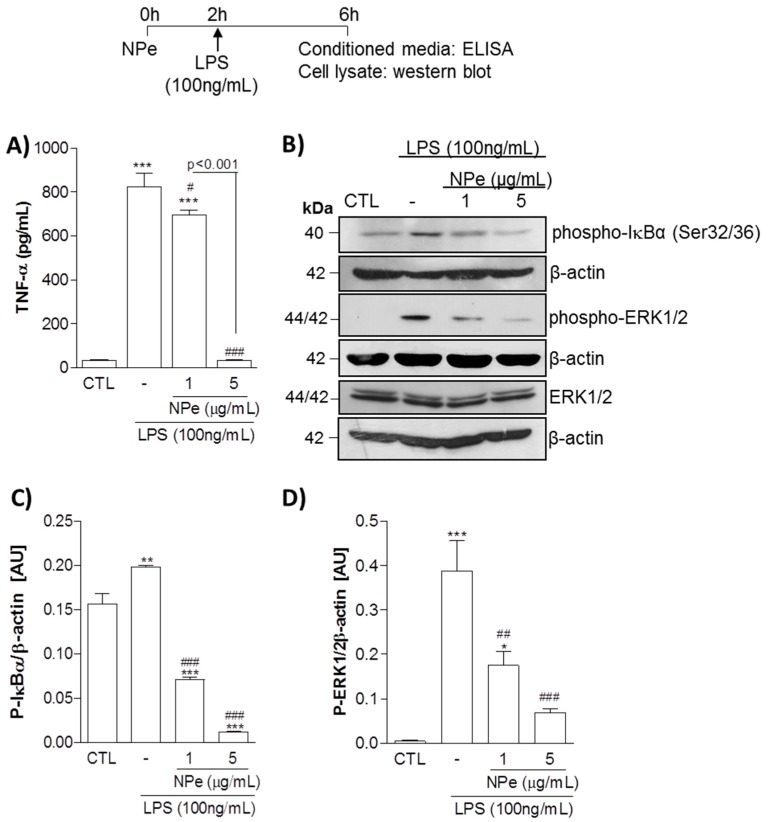
Effect of NPe on LPS-stimulated THP-1 cells assayed at the non-toxic doses. Human promonocytoid cell line THP-1 was transferred to a 96-well plate at a density of 50,000 cells per well, differentiated into macrophages and serum-deprived with FBS 0.5% for 16 h. Subsequently, cells were either left untreated, or treated with NPe at different concentrations (1 and 5 μg/mL) for 2 h, and thereafter with or without LPS (100 ng/mL) for a further 4 h. (**A**) TNF-α release was measured in the supernatant. Data are shown as the mean ± SEM of three wells in each group. Experiments were performed at least 3 times with similar results. *** *p* < 0.001 when compared to the control group. ^#^ *p* < 0.05 and ^###^ *p* < 0.001 when compared treated to untreated cells challenged with LPS. The statistical difference between the 2 doses of NPe used is indicated in the graph; (**B**) Phosphorylated IκB-α and ERK1/2, as well the total form of ERK, were assessed by western blot analysis. Blots were normalized with β-actin and are representative of three independent experiments using pooled cells from at least four wells; (**C**,**D**) Densitometry data for phosphorylated ERK1/2 and IκB-α are represented graphically. * *p* < 0.05, **p < 0.01, and *** *p* < 0.001 when compared to the control group. ^##^ *p* < 0.01 and ^###^ *p* < 0.001 when compared cells challenged with LPS treated or not with NPe. All experiments were performed at least 3 times (n = 3 in each group) with similar results.

Next, we assessed the anti-TNF-α activity of NPe over THP-1 macrophages stimulated with LPS (Figure 4A). Pre-treatment of cells with NPe 2 h before stimulation with LPS significantly reduced the production of TNF-α at the two tested concentrations. The reduction in TNF-α production by NPe was concentration-dependent, with complete prevention of cytokine production observed for NPe at 5 μg/mL. In line with these observations, NPe attenuated the activation of ERK pathway induced by LPS, whereas total ERK levels did not change (Figure 4B). Once again, the effect of NPe in preventing cell activation by LPS was -dependent. Hence, the increased phosphorylation of IκB-α induced by LPS was reduced by NPe at 1 μg/mL and almost abolished by NPe at a higher concentration (5 μg/mL). In sum, NPe at non-toxic doses opposed the inflammatory effects induced by LPS stimulation, namely, production of the inflammatory cytokine TNF-α and activation of NF-κB and ERK pathways.

### 3.3. Pre-Treatment of LPS-Inflamed Mice with NPe Reduces Neutrophil Accumulation and Inflammatory Markers

**Figure 5:**
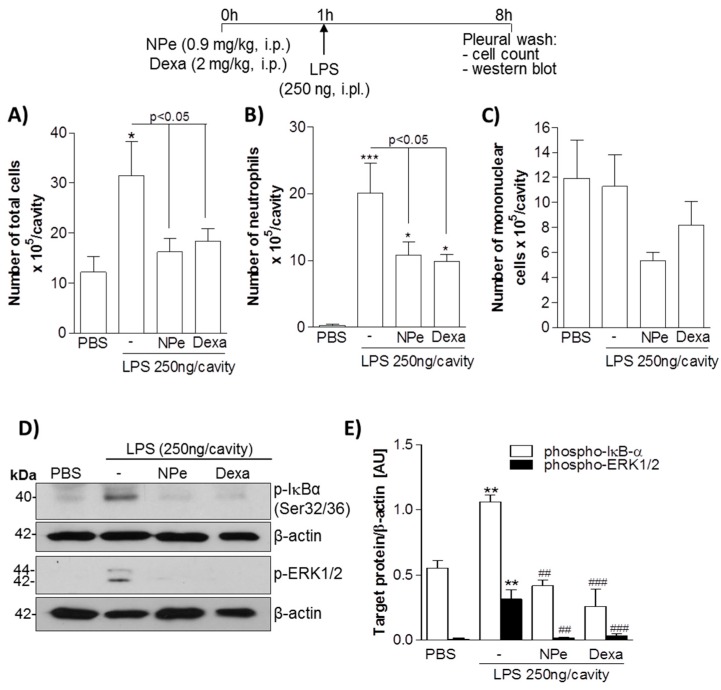
Effect of pre-treatment of mice with NPe on LPS-induced pleurisy. Mice received an injection of equimolar dose of NPe (0.9 mg/kg, i.p.), dexamethasone (2 mg/kg, i.p.), or vehicle (2% DMSO/PBS). After 1 h, mice were challenged with LPS (250 ng/cavity, i.pl.). Cells from pleural cavity were processed for total (**A**) and differential (**B**,**C**) cell counts. The pleural cellularity was expressed as number of leukocytes per cavity, and is shown as mean ± SEM of at least four mice in each group. * *p* < 0.05 and *** *p* < 0.001 when compared with unchallenged mice (that did not receive LPS injection). Statistical differences between LPS-injected mice treated or not with NPe are indicated in the graph; (**D**) Inflammatory markers were analyzed for the whole extract of cells harvested from the pleural cavity. Blots were normalized with β-actin and are representative of three independent experiments using pooled cells from at least four mice; (**E**) Densitometry data for phosphorylated ERK1/2 and IκB-α are represented graphically. ** *p* < 0.01 when compared with mice that did not receive LPS injection. ^##^ *p* < 0.01 and ^###^ *p* < 0.001 when comparing LPS-injected mice treated or not with NPe.

Next, we evaluated whether NPe could operate in vivo to prevent neutrophil accumulation induced by LPS. Therefore, we treated mice with an intraperitonial (i.p.) injection of NPe, followed by an intrapleural (i.pl.) inflammatory challenge with 250 ng of LPS. Dexamethasone was used as a positive control. As expected, i.pl. injection of LPS induced an influx of leukocytes into the pleural cavity of mice (Figure 5A). Differential cell counts revealed that cells migrated into the cavity were mainly neutrophils (Figure 5B), with no significant increase in number of mononuclear cells (Figure 5C). The analysis of pleural cellularity showed that pre-treatment with NPe decreased neutrophil accumulation in the pleural cavity 7 h after LPS injection when compared to untreated mice (Figure 5B). Remarkably, equimolar doses of NPe and dexamethasone, had similar effectiveness in reducing neutrophil accumulation in the pleural cavity of LPS-challenged mice. As previously demonstrated [32,33], LPS injection markedly induced activation of pro-inflammatory pathways in pleural cells, as observed by the increased phosphorylated forms of ERK1/2 and IκB-α, proteins associated with neutrophil survival and consequent accumulation. In this study, we observed that NPe prevented the activation of both pathways, as demonstrated by the return of phospho-ERK and phospho-IκB-α levels to basal levels, as compared to the unchallenged group. Again, NPe was as effective as dexamethasone in inhibiting ERK1/2 and NF-κB pathways (Figure 5D,E). Overall, NPe acted in a similar manner to dexamethasone to reduce neutrophil accumulation and prevent activation of neutrophil pro-survival pathways in response to LPS.

## 4. Discussion

Inflammation occurs as a result of recognition of foreign bodies or self-antigens by patrolling cells present in tissues, followed by increased vascular permeability, leukocyte recruitment and release of pro-inflammatory mediators [16,17] . Recognition of microbial products, such as LPS from membrane of Gram-negative bacteria, by toll-like receptors and other pattern-recognition receptors, initiates the production of pro-inflammatory cytokines including TNF-α, IL-1 and IL-6, as well as a number of chemokines. Although an efficient inflammatory response is crucial for host defense, uncontrolled inflammation leads to chronic or autoimmune diseases [18]. Since deregulated inflammation is usually associated with excessive cytokine production, inhibiting cytokine action or production represent important pharmacological strategies for resolving acute inflammation. In addition to the reduction of chemokine and cytokine gradients, the termination of inflammation, the so-called resolution of inflammation, depends on down-regulation of survival pathways, apoptosis of granulocytes and their effective removal by macrophages [17]. In this study, we explored the anti-inflammatory effects of NPe and the underlying mechanisms both in vitro and in vivo.

NPe and its derivatives have been found to exert various biological effects, including anti-edema [35], vasorelaxant [13], antiplatelet [36,37] , anti-inflammatory [35,38], and anticancer activities [39,40]. Despite the several therapeutic actions described for NPe derivatives, they are also recognized to reduce cell viability [37,40,41]. In many cases, apoptosis, a programmed cell death, was identified as the mechanism of cell death induced by NPe and its derivatives [40,41,42]. Apoptosis-inducing cytotoxic activity associated with caspase-3 activation was also identified for hybrid analogs of NPe, nitrovinylstilbene and flavanone scaffolds in breast cancer cells [41]. Neutrophil apoptosis is essential for the termination of inflammation [17], and the pro-apoptotic effect of NPe could be advantageous in inflammatory contexts. On the other hand, monocytes and macrophages are crucial for the resolution of inflammation and should be preserved from cell death. With this in mind, we initially assessed the non-toxic concentrations of NPe on monocytes and macrophages. Here, we identified that NPe did not alter cell viability of murine and human macrophages in concentrations lower than or equal to 1 μg/mL and 5 μg/mL, respectively.

The anti-inflammatory potential of nitroderivatives has been previously demonstrated [35,38]. For instance, the derivative 3,4-methylenedioxy-β-nitrostyrene exhibited inhibitory effects on ATPase and decreased inflammasome activation [38]. This β-nitrostyrene derivative also exhibited suppressed β1-integrin activation resulting in suppression of breast cancer cell adhesion and migration [39]. It is known that integrin-mediated cell adhesion and migration are not only important for cancer metastasis, but also for leukocyte recruitment during inflammation [43]. Thus, the suppression of β1-integrin activation by β-nitrostyrenes could also interfere with leukocyte migration, thus acting as an anti-inflammatory. NPe derivatives have also been demonstrated to inhibit the TNFα/NFκB signaling pathway in MCF-7 human breast cancer cells [44]. Furthermore, several nitroderivatives, including NPe itself, reduced edema and myotoxic activity provoked by PLA_2_ purified from snake venom [35]. In this study, we found that, in non-toxic concentrations, NPe inhibited TNF-α release by LPS-stimulated THP-1 human macrophages, and reduced the levels of phosphorylated ERK and IκB-α. These results indicate the potential anti-inflammatory properties of NPe, since it significantly reduced macrophage activation by LPS. Future studies might investigate whether the altered levels of TNF-α protein, observed in this study, are a consequence of transcriptional regulation of TNF-α gene expression. Changes in gene expression by NPe are likely to be observed, as suggested by the decreased levels of phospho-ERK in treated macrophages, a MAPK involved in gene expression of pro-inflammatory cytokines. In order to provide complementary observations, future studies will also need to address the effect of NPe on other pro-inflammatory cytokines (e.g., IL-1β and IL-6), as well as other important pro-inflammatory mediators (e.g., prostaglandins and nitric oxide) and pro-inflammatory pathways in macrophages, such as cyclooxygenase-2, the main enzyme in the arachidonic acid cascade responsible for eicosanoid production [45]. The regulation of ERK1/2 phosphorylation being associated to the reduced protein expression of TNF-α by NPe suggests that this nitroderivative regulates the downstream transcription factor AP-1, which must be confirmed in future. Indeed, we cannot exclude that NPe can also have an inhibitory effect on other MAPKs, such as JNK and p38, in addition to the observed effect on ERK1/2 phosphorylation. This study also opens new avenues of research for better understanding the effect of NPe on interferon regulatory factor (IRF)-signaling, another important signaling pathway in macrophages.

Equally important was to expand these in vitro analyses by investigating the efficacy of NPe on acute inflammation in a murine model of pleurisy induced by LPS. In this study, we observed that NPe was as effective as an equimolar dose of dexamethasone in reducing neutrophil accumulation in the pleural cavity after LPS challenge. Indeed, we noticed that the levels of phospho-ERK and phospho-IκB-α were decreased after exposure to NPe. These observations indicate that the inhibition of NF-κB and ERK1/2 pathways may contribute at least in part, to the anti-inflammatory actions observed for NPe in this study.

In summary, NPe showed anti-inflammatory properties both in vitro and in vivo. In vitro, it prevented macrophage activation, as indicated by the abolished production of pro-inflammatory cytokine and reduced activation of the NF-κB and ERK pathways. In an experimental model of acute inflammation, pre-treatment with NPe was as effective as dexamethasone in reducing neutrophil accumulation and preventing the activation of NF-κB and ERK1/2 pathways. These results indicate the potential anti-inflammatory properties of NPe by interfering with leukocyte migration and activation of pro-inflammatory pathways.
